# Acceptance and disparities of PET/CT use in patients with esophageal or gastro-esophageal junction cancer: Evaluation of mature registry data

**DOI:** 10.3389/fnume.2022.917873

**Published:** 2022-09-16

**Authors:** Vaibhav Gupta, Roshini Kulanthaivelu, Ur Metser, Claudia Ortega, Gail Darling, Natalie Coburn, Patrick Veit-Haibach

**Affiliations:** ^1^ Department of Surgery, University Health Network / Mount Sinai Hospital, University of Toronto, Toronto, ON, Canada; ^2^ Joint Department of Medical Imaging, University Health Network, Mount Sinai Hospital and Women’s College Hospital, University of Toronto, Toronto, ON, Canada; ^3^ Department of Surgery, Sunnybrook Health Sciences Centre, University of Toronto, Toronto, ON, Canada

**Keywords:** esophagus, cancer, PET/CT, disparities, Canada

## Abstract

**Background/rationale:**

PET/CT plays a crucial role in esophageal (EC) and gastroesophageal junction cancer (GEJ) diagnosis and management. Despite endorsement in clinical guidelines, variation in acceptance of PET/CT exists. The aim of this study was to assess the early use of PET/CT among EC and GEJ patients in a regionalized setting and identify factors contributing to disparity in access.

**Materials and methods:**

Retrospective cohort study of adults with EC or GEJ between 2012 and 2014 from the Population Registry of Esophageal and Stomach Tumours of Ontario and Ontario Health (Cancer Care Ontario). Receipt of PET/CT and relevant demographics were collected, and statistical analysis performed. Continuous data were analysed with t-tests and Wilcoxon rank sum test. Categorical data were analysed with chi-square test. Kaplan–Meier methods were used to estimate median survival.

**Results:**

Fifty-five percent of patients diagnosed with EC or GEJ between 2012 and 2014 received PET/CT (1321/2390). Eighty-four percent of patients underwent surgical resection (729/870), and 80% receiving radical treatment (496/622) underwent PET/CT. The use of PET/CT increased from 2012 to 2014. Male patients received more PET/CT than females (85% vs.78% *p* < 0.001).

Median survival for the overall cohort was 11.1 months, 17.2 vs. 5.2 months among those who did and did not receive PET/CT and 35 vs. 27 months among the surgical cohort (*p* = 0.16).

**Conclusions:**

We found that PET/CT use increased from 2012 to 2014 and that the majority of EC/GEJ patients being considered for curative therapy received PET/CT. There were also gender disparities identified. PET/CT appears to confer a potential survival benefit in our study, although our assessment is limited. Our findings may serve as learned lessons for other new imaging modalities, new indications for PET/CT or even for the introduction of new radiopharmaceuticals for PET/CT.

## Introduction

Esophagogastric cancer is the seventh most common cancer in the world and sixth leading cause of cancer death worldwide. It have a high morbidity and mortality, affecting up to 2400 Canadians each year (1). Squamous cell carcinoma (SCC) is still most common histological type worldwide but in North America and Europe there has been increased rate of adenocarcinoma in the last decades. Accurate staging is integral to successful management, with surgical resection, the main form of curative therapy. The prognosis of esophageal cancer (EC) has generally improved over the years, however it remains relatively low even in patients with early-stage disease treated with curative intent. FDG-PET/CT plays a crucial role in the evaluation of EC and gastroesophageal junction cancers (GEJ) due to increased sensitivity for the detection of metastatic disease, as demonstrated in studies dating back to 1997 (2–7). PET/CT also permits functional tumour assessment, with an emerging evidence-base supporting its use in evaluating response to neoadjuvant therapy (5–11) and detection of recurrent disease (1–3, 5–7).

PET/CT has been successfully incorporated into multiple clinical guidelines from the American NCCN (12), European Society of Medical Oncology (13), the British NICE (14), Royal College of Radiology (15) and British Society of Gatroenterology (16). PET/CT is universally recommended for initial staging of EC and GEJ patients being considered for radical therapy, with an increasing role for PET/CT post neoadjuvant therapy and in the detection of recurrent disease (12–16).

In Canada, PET/CT was initially recommended by Ontario Health-Cancer Care Ontario (OH-CCO) for initial staging of EC and GEJ patients being considered for curative therapy, and performed under a research registry between 2009 and 2011 (17). The purpose of this was to demonstrate sufficient clinical benefit so that the government would include PET/CT for EC and GEJ in the publicly funded Ontario Health Insurance Plan (OHIP). On April 1, 2012 PET/CT was subsequently included in the schedule of benefits for “baseline staging assessment of those patients with esophageal/gastroesophageal junction cancer being considered for curative therapy”, “post pre-operative/neoadjuvant therapy”, and for “restaging of EC/GEJ with locoregional recurrence” (18).

Despite the successful incorporation of PET/CT into multiple guidelines, the acceptance of PET/CT in clinical practice is variable. Healthcare disparities exist and the causes are complex. The Canadian population is served by a government-based, publicly funded healthcare system composed of an “interlocking set of ten provincial and three territorial health systems” (19). The province of Ontario serves a population of approximately 14 million over an area of 1 million km^2^, with the highest population in Toronto (20). The delivery of uniform provincial healthcare in this setting is challenging, particularly in the context of centralisation of specialist cancer care. PET/CT across Ontario is currently delivered by 40 different providers across 10 different institutions and EC/GEJ managed in both community and tertiary settings with surgery conducted at academic tertiary centres.

The primary aim of our study was to evaluate the early acceptance of PET/CT across the province among patients with EC/GEJ, and see if there were any potential factors contributing to disparity in access to this valuable imaging tool.

## Methods

### Study design and setting

We performed a retrospective cohort study of adults diagnosed with EC or GEJ in Ontario, Canada (population 13.6 million). Patients were identified in the Population Registry of Esophageal and Stomach Tumours of Ontario (PRESTO) database, which is based on the Ontario Cancer Registry (OCR). The OCR captures 96% of cancer diagnoses in Ontario. A separate sub cohort of prospectively identified patients with EC or GEJ undergoing PET/CT was obtained from OH-CCO for comparison.

Ethical approval for this study was obtained from the Sunnybrook Health Sciences Centre Research Ethics Board. Data confidentiality and privacy policies from Sunnybrook and IC/ES were adhered to throughout the study.

### Study participants

All patients with esophageal cancer (overall cohort) diagnosed between 2012 and 2014 in Ontario were identified from the PRESTO database using relevant topography, histology, and surgical codes (see Supplementary Table S1). A subgroup of patients undergoing surgery (resected cohort) were analysed separately with additional information/variables of interest detailed below. Patients were excluded if they were less than 18 years of age, not eligible for government health insurance, or had a cancer type other than adenocarcinoma or squamous cell carcinoma.

The number of patients with potentially curative EC/GEJ were identified separately from OH-CCO from 2012 to 2017. No demographic information or follow-up data was available for this sub cohort of patients.

### Data sources

Data for the EC/GEJ cohort from the PRESTO database were linked from the following sources (see Supplementary Table S2): OCR, Ontario Health Insurance Plan database, Canadian Institute for Health Information Discharge Abstract Database, and the Registered Persons Database. These datasets were linked using unique encoded identifiers and analyzed at IC/ES, a non-profit independent research organization (www.ices.on.ca).

The subcohort of patients obtained directly from OH-CCO was linked from Activity Level Reporting (ALR) and Canadian Institute of Health Information-National Ambulatory Care Reporting System metadata (CIHI-NACRS), in addition to those listed above.

### Variables of interest

The main exposure of interest was receipt of PET/CT during the patient's treatment journey (yes/no). This was defined as a submitted OHIP claim for interpretation of a PET/CT for the patient at any point during the study period.

The demographics of interest for the overall cohort included age, sex, tumour site (esophagus or GEJ), histology (adenocarcinoma or squamous cell carcinoma), rural residence (yes/no), diagnosis year, receipt of surgery (yes/no), and median survival. Additional variables of interest for patients undergoing surgery included comorbidity (defined using the Johns Hopkins Adjusted Diagnosis Groups methodology into low, moderate, and high), major postoperative complications (defined as cerebrovascular accident, myocardial infarction, acute kidney injury, respiratory failure, pneumonia, airway compromise, pulmonary embolism, deep vein thrombosis, cardiac arrest, shock, sepsis, or procedure-related complications, such as bleeding or anastomotic leak), and use of chemotherapy or radiotherapy preoperatively or postoperatively. Follow-up data were evaluated up until November 2016.

2-[18F] FDG PET/CT imaging was generally obtained based on the respective institutional protocol. As a minimum, patients are always instructed to avoid exercise for 24 h and fast for 6 h before the examination. Patients receive a weight adapted IV injection of FDG (typically a range of 250–550 MBq). Iodinated oral contrast material was partly administered for bowel opacification; generally no intravenous iodinated contrast is administered as a standard.

### Statistical methods

Descriptive statistics were used to describe baseline demographics and compare patients who did and did not receive PET/CT. Continuous data were presented with means and standard deviations and analyzed using independent sample t-tests if normally distributed; non-normally distributed data were presented with medians and interquartile ranges and analyzed using the Wilcoxon rank sum test. Categorical data were presented as frequencies and proportions; comparisons were performed using the chi-square test. Kaplan-Meier methods were used to estimate median survival. A *p-*value <0.05 was considered statistically significant. All tests were 2-tailed and were performed using the SAS software application (version 9.2: SAS Institute, Cary, NC, United States).

## Results

### Overall cohort

18F (FDG) PET/CT has a central role in staging as well treatment response assessment in a neo-adjuvant setting as well as post-surgical setting. It also is one of the central imaging procedures for evaluation of oesophageal/gastro-oesophageal cancers in several guidelines. Recurrence detection after treatment in various malignancies including esophagogastric cancers and the respective value in prognostication in this patients population has been described in many publications since it's introduction in clinical routine.

Of 2390 patients across Ontario diagnosed with EC or GEJ from 2012 to 2014, 1321 patients received a PET/CT scan (55%). Consequently 1069 patients underwent therapy without PET/CT staging (45%). One hundred and fifty patients received two or more PET/CT scans during their treatment journey (6%). The number of patients undergoing PET/CT increased from 2012 to 2014, increasing from 349 (52.7%) to 479 (56.4%). Table 1 shows the characteristics of patients who did and did not receive PET/CT scan.

**Table 1:** Cohort demographics stratified by receipt of PET/CT.

Variable		No PET *n* = 1069	Had PET *n* = 1321	Total *n* = 2390	*p*-value
Age	Mean ± SD	68.91 ± 13.25	64.67 ± 10.79	66.57 ± 12.13	**<0.**001
Sex	Female	300 (53.7%)	259 (46.3%)	559 (100.0%)	**<0.**001
	Male	769 (42.0%)	1,062 (58.0%)	1,831 (100.0%)	
Site	GEJ	503 (52.3%)	458 (47.7%)	961 (100.0%)	**<0.**001
	Esophagus	566 (39.6%)	863 (60.4%)	1,429 (100.0%)	
Histology	AC	757 (43.2%)	995 (56.8%)	1,752 (100.0%)	**<0.**001
	SCC	182 (40.7%)	265 (59.3%)	447 (100.0%)	
	Other	130 (68.1%)	61 (31.9%)	191 (100.0%)	
Rural residence	N	881 (44.6%)	1,093 (55.4%)	1,974 (100.0%)	0.803
	Y	188 (45.3%)	227 (54.7%)	415 (100.0%)	
Year	2012	313 (47.3%)	349 (52.7%)	662 (100.0%)	0.296
	2013	386 (43.9%)	493 (56.1%)	879 (100.0%)	
	2014	370 (43.6%)	479 (56.4%)	849 (100.0%)	
Surgery	No	963 (57.9%)	699 (42.1%)	1,662 (100.0%)	**<0.**001
	Yes	106 (14.6%)	622 (85.4%)	728 (100.0%)	

### Demographics

There were some important demographic differences in the receipt of PET/CT identified among the cohort of EC/GEJ patients (Table 1). Most notably, males underwent statistically significant more PET/CT than females 85% (1062/1321) vs. 78% (259/1321) (*p* < 0.001). Patients with EC were referred more than patients with GEJ cancers (89% (458/1321) vs. 77% (458/1321)) (*p* < 0.001). There was no statistically significant difference in the receipt of PET/CT among patients from a rural vs. urban residence (*p* = 0.803). Patients with higher number of additional comorbidities were referred statistically significantly less for PET/CT for staging (84% for low (194/729), 86% for moderate (398/729), and 77% for high (137/729) comorbidity) (*p* < 0.001). There was no difference in PET/CT use between adenocarcinoma and squamous cell carcinoma histology (*p* > 0.05).

Among the cohort of EC/GEJ patients that underwent resection, PET/CT use also appeared to be higher among those patients who received neoadjuvant therapy. For example, 88% of patients receiving preoperative chemotherapy and 90% of patients receiving preoperative radiotherapy received a PET/CT scan, with most patients receiving combined chemo-radiation. In comparison, 80% of patients receiving postoperative chemotherapy and 78% of patients receiving postoperative radiotherapy received a PET/CT scan.

### Resected cohort

Of 870 patients undergoing surgery for EC/GEJ from 2012 to 2014, 729 (84%) had a PET/CT scan completed (Table 2). This increased from 73% in 2012 to 91% in 2014. We assume that PET/CT was found increasingly helpful after it was being offered to patients under the public schedule of benefits and that referring physicians increasingly relied on this imaging biomarker method for staging.

**Table 2:** Among resected patients, demographics stratified by receipt of PET/CT.

Variable		No PET *N* = 141	Had PET *N* = 729	TOTAL *N* = 870	*p*-value
Age	Mean ± SD	63.82 ± 11.58	62.83 ± 10.39	62.99 ± 10.59	0.31
Sex	Female	34 (21.9%)	121 (78.1%)	155 (100.0%)	**0** **.** **033**
	Male	107 (15.0%)	608 (85.0%)	715 (100.0%)	
Site	Esophagus	58 (11.5%)	446 (88.5%)	504 (100.0%)	**<0.**001
	GEJ	83 (22.7%)	283 (77.3%)	366 (100.0%)	
Histology	AC	120 (16.3%)	616 (83.7%)	736 (100.0%)	0.86
	SCC	21 (15.7%)	113 (84.3%)	134 (100.0%)	
Comorbidity	Low	36 (15.7%)	194 (84.3%)	230 (100.0%)	**0** **.** **031**
	Moderate	65 (14.0%)	398 (86.0%)	463 (100.0%)	
	High	40 (22.6%)	137 (77.4%)	177 (100.0%)	
Major postoperative complication		49 (16.6%)	247 (83.4%)	296 (100.0%)	0.84
Rural residence		22 (14.8%)	127 (85.2%)	149 (100.0%)	0.60
Year	2012	81 (27.4%)	215 (72.6%)	296 (100.0%)	**<0.**001
	2013	35 (12.4%)	248 (87.6%)	283 (100.0%)	
	2014	25 (8.6%)	266 (91.4%)	291 (100.0%)	
Preoperative chemotherapy		68 (12.5%)	478 (87.5%)	546 (100.0%)	**<0.**001
Preoperative radiotherapy		42 (9.6%)	396 (90.4%)	438 (100.0%)	**<0.**001
Postoperative chemotherapy		47 (20.3%)	184 (79.7%)	231 (100.0%)	**0** **.** **046**
Postoperative radiotherapy		27 (22.0%)	96 (78.0%)	123 (100.0%)	0.06

AC, adenocarcinoma; SCC, squamous cell carcinoma.

### Ontario health-cancer care ontario cohort

**Figure 1:**
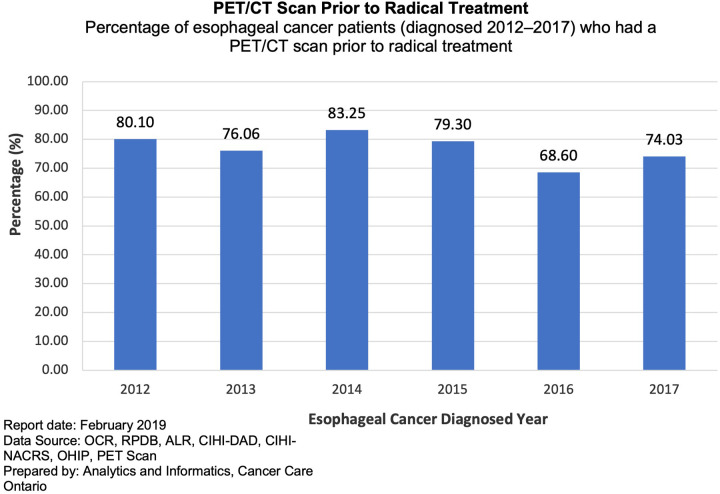
Percentage of esophageal cancer patients who had a PET/CT scan prior to radical treatment from 2012 to 2017.

Figure 1 illustrates the number and percentage of EC/GEJ patients receiving a PET/CT prior to radical treatment from 2012 to 2017 as per OH-CCO. Of 622 patients considered for radical treatment from 2012 to 2014, 80% received a PET/CT. It illustrates an early increase in both the numbers of PET/CT among EC/GEJ patients receiving radical treatment from 2012 to 2014, rising from 80% to 83%. OH-CCO's target for PET/CT in EC/GEJ is >90%, however, the % of PET/CT among patients receiving radical treatment lies below this threshold, and appears to plateau between 2012 and 2017 (Figure 1).

### Efficacy of PET/CT

Median survival for the overall cohort was 11.1 months. Among patients who received a PET/CT scan, survival was 17.2 months. Patients who did not receive a PET/CT scan had a median survival of 5.2 months (*p* < 0.001).

Median Survival among the resected cohort of patients receiving PET/CT was 35 months and 27 months for those who did not (*p* = 0.16).

**Figure 2:**
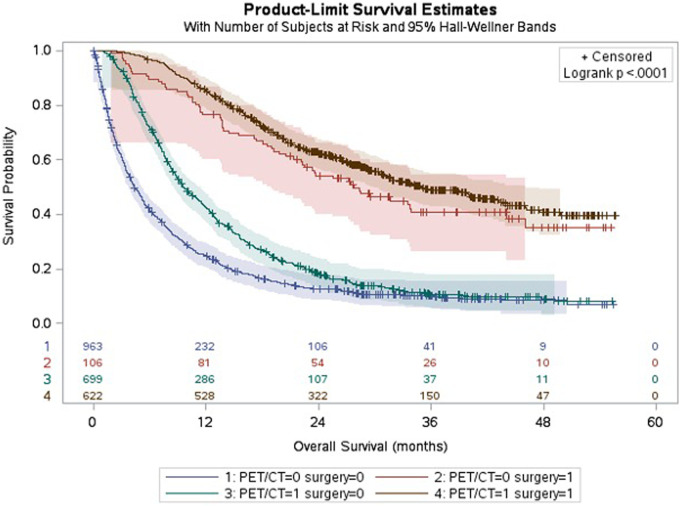
Kaplan-Meier curves showing survival stratified by receipt of PET/CT and surgery. Legend: **Median survival**, all patients: 11 months; −PET − Surg: 4 months; −PET + Surg: 27 months; +PET − Surg: 9 months; +PET + Surg: 35 months.

Figure 2 shows the different survival trajectories stratified by patients who did or did not receive a PET/CT scan and did or did not receive surgery. A clear, (expected) statistically significant separation is demonstrated between patients who underwent/did not undergo surgery. Also, a clear initial separation can be seen between patients who did/ did not undergo PET/CT in the first 2 years in both groups (resected and non-resected). There was however no statistical difference found in terms of long-term survival difference.

## Discussion

There are few descriptive studies in the literature to date evaluating and describing the early acceptance of PET/CT and potential disparities within a publicly funded healthcare system, in a specific cancer indication with a large dataset. Our study found that there is a delay until sufficient acceptance of PET/CT is achieved, there exists potential gender disparity and, finally, improved short-term survival among patients undergoing PET/CT compared to patients who are not.

PET/CT is important for the successful management of patients with EC and GEJ. There is a strong evidence base supporting the routine use of PET/CT for initial staging among patients being considered for curative treatment, and an emerging role in assessment post neoadjuvant therapy. Although PET/CT has been successfully incorporated into several guidelines, the adoption of PET/CT into clinical practice can initially prove slow, as illustrated in our study. In Ontario, PET/CT for EC/GEJ was initially performed under a research registry among patients considered for curative/radial treatment, in order to prove a clinical benefit. This was so that the government would fund PET/CT, despite the presence of a strong evidence base in the published literature. This initial apparent hesitation to pursue PET/CT, may therefore be related to referring physicians viewing PET/CT as experimental or difficult to obtain due to additional paperwork required and the perceived delay in scheduling due to the centralisation of PET/CT services.

Although only 55% of patients in our study underwent PET/CT (1321/2390), 84% of patients who underwent surgical resection received PET/CT (729/870). This finding was confirmed within the OH-CCO sub-cohort where 80% of patients receiving radical treatment underwent PET/CT. The difference observed when compared with data obtained from the PRESTO database is likely due to the difference in data sources, where the OH-CCO data represents a subcohort of prospectively acquired data among patients who were being considered for radical treatment (both surgical and non-surgical). The role of PET/CT among patients with extensive metastatic, potentially unresectable, stage IV disease demonstrated on standard cross-sectional imaging is limited and OH-CCO guidelines recommend the use of PET/CT among patients who are considered “candidates for curative therapy” (18). The increased use of PET/CT among the surgical and OH-CCO cohort, demonstrated in our study, is therefore concordant with current OH-CCO guidelines. While there might also be value of PET/CT surveillance in patients with palliatively treated disease, this is beyond the scope of this study.

### Demographic differences in receipt of PET/CT

Currently, there are very few studies in the published literature exploring disparity in PET/CT utilisation and none are available from a public or regionalized jurisdiction. A study from 2012 by Onega et al. identified higher use of PET/CT in areas of greater medicare spending and among white patients of higher socioeconomic groups (21). Additionally receipt of PET/CT in lung cancer has been identified as a source of racial disparity in the US, where a small number of studies have identified that non-white patients were less likely to undergo guideline-recommended PET/CT or CT imaging at lung cancer diagnosis (22–24). A higher incidence of PSMA PET/CT for prostate cancer has also been seen among non-Hispanic white patients (25). There are however no studies exploring disparity in access and usage of PET/CT among EC and GEJ patients, outside America. Even though we did not have racial information on our sample and that the Canadian healthcare system is known to be significantly more equitable compared to the US and other countries with dual/multi-tier health care systems, we found certain differences and disparities in other variables (i.e. sex) regarding PET/CT access.

EC/GEJ has a male preponderance, constituting 74% of patients across Canada from 1992 to 2010 (26), with a higher rate of adenocarcinoma compared with squamous cell carcinoma (7, 26). The results of our study confirm this finding with 77% male patients (1831/2390), and 73% patients with adenocarcinoma (1752/2390). However, this male preponderance in incidence does not account for the statistically significantly higher rate of PET/CT use seen among male vs. female patients [85% (1062/1321) vs. 78% (259/1321)]. As PET is indicated among patients considered for curative treatment, this may reflect bias at a clinical level, with more male patients being considered for surgery and subsequently referred for PET/CT. The cause for this disparity however, is not clear. Similar findings were however shown in a study by Scholttmann et al. from 2020 which identified that female patients were less likely to undergo surgery compared with males (27).

**Figure 3:**
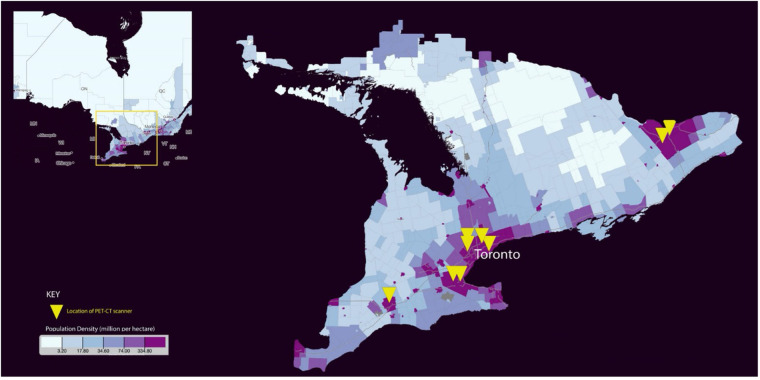
Population density distribution and location of PET/CT scanners across Ontario [*Map created from Statistics Canada* (30)].

The distribution of PET/CT across the province of Ontario mirrors the population density (Figure 3). This is similar to findings from Onega et al. (21), where the majority of PET/CT was performed in urban areas with higher healthcare spending. Despite this regionalisation of PET/CT services, we did not however identify any geographical disparity in access to PET/CT. The majority of patients within our study resided in an urban area (83%, 1974/2390), with no significant difference in the use of PET/CT among patients from an urban vs. rural residence.

The reasons for an increased rate of PET/CT being used among esophageal vs. GEJ malignancies, and a reduced rate of PET/CT among patients with greater co-morbidities undergoing less PET/CT may be related to clinician patient selection. Clinical debate exists regarding GEJ cancers, and whether to treat in the same manner as esophageal or gastric cancers, despite emerging evidence that PET/CT has a significant impact on staging and subsequent evaluation in gastric cancer (28) and patients with greater co-morbidities are less likely to be considered good candidates for surgical resection. These findings may however also represent a potential access bias, since the decision about curative therapy should ideally always be made after complete staging and not only based on co-morbidities.

### Efficacy of PET/CT

The reported 5-year survival rate for patients with EC is 19.9% or 24 months (29). The median survival for our entire cohort was 11.1 months. A lower median survival (5.2 months) and higher rates of comorbidity were seen among patients who did not receive PET/CT compared with those who did (17.2 months). Those who did not receive PET/CT likely reflect patients with extensive metastatic disease at presentation and/or who were considered poor surgical candidates and would therefore not routinely necessitate staging with PET/CT. We know from our data that the majority of PET/CT was performed among the cohort of patients who underwent surgery (88%), therefore this improved median survival with PET/CT likely reflects patient selection bias.

When comparing the median survival among the surgical cohort that did not receive PET/CT (27 months) and did receive PET/CT (35 months), we do however see an apparent small improvement in short-term survival, although not statistically significant long term. The lost survival benefit can likely be explained with recurrences which are often seen in the early period after curative surgery.

In addition to staging, the information from PET/CT can be used to guide radiotherapy planning in both the neoadjuvant and curative setting. As surgery is usually provided at a tertiary level, the patient cohort receiving PET/CT may also potentially experience higher rates of palliative chemo-or radiotherapy due to management at these centres, potentially explaining the improved median survival seen in the cohort of patients who did not undergo surgery but received PET/CT (9 vs. 4 months). While this finding certainly reflects (at last partly) a Will Rogers Phenomenon (stage migration), we have implicitly made the assumption that if patients did not get surgery, their treatment was not planned with curative intent since only a minority of patients get curative radio-chemotherapy for this disease.

### Limitations

This is a population-level registry descriptive study assessing the early acceptance of PET/CT following approval for funding in a public health care setting among patients with EC or GEJ between 2012 and 2014. As such, the study suffers from common limitations related to the secondary source of information. For example, several factors are missing or only partially available. While we evaluated rural vs. urban residence (as a marker for access), we did not evaluate further factors regarding the patients' socioeconomic status such as income or race and ethnicity, which have been identified as factors contributing to disparity in care in several other cancer entities. While the timeframe of our evaluation is obviously not current, this was chosen on purpose since we wanted to evaluate the acceptance within the introduction phase of an imaging modality for a specific disease to learn about potential disparities. Also wanted to report on the respective correlated survival which required a prolonged period of observation. This project was however additionally delayed due to the pandemic. Lastly, the here found disparities in in healthcare access might not be ubiquitously present in other western healthcare system. This might be especially relevant for health care systems with a dual tier insurance system where more variable access to health care service is possible.

## Conclusions

The study evaluated and described the early acceptance of PET/CT for EC and GEJ in a regionalized public health care system. We found that the use of PET/CT increased from 2012 to 2014, and that the majority of patients undergoing potentially curative therapy received staging PET/CT as per OH-CCO guidelines. Gender disparity in access to PET/CT was however identified, with a statistically significant higher number of males vs. females undergoing PET/CT.

Finally, although limited, our study infers a potential survival benefit in patients undergoing PET/CT both with and without surgery. These findings may serve as learned lessons for other new imaging modalities, new indications for PET/CT or even for the introduction of new radiopharmaceuticals for PET/CT.

## Data Availability

The raw data supporting the conclusions of this article will be made available by the authors, without undue reservation.

## Author disclaimer

This overall study was supported by ICES, which is funded by an annual grant from the Ontario Ministry of Health and Long-Term Care (MOHLTC). Parts of this material are based on data and/or information compiled and provided by the Canadian Institute for Health Information (CIHI) and by Ontario Health-Cancer Care Ontario (OH-CCO). The analyses, opinions, results and conclusions reported in this paper are those of the authors and are independent from ICES, MOHLTC, CIHI, and CCO. No endorsement by any of these organizations is intended or should be inferred.
