# Supplementary material for: Acceptance and disparities of PET/CT use in patients with esophageal or gastro-esophageal junction cancer: Evaluation of mature registry data
Source: Front Nucl Med. 2022 Sep 16;2:917873. doi: 10.3389/fnume.2022.917873 (PMC11440829; doi:10.3389/fnume.2022.917873)
Supplement: Supplementary file 1 [file DataSheet1.docx]

## Supplemental Data (Online Only)

**Supplemental Data 1, Table**: ICD-O-3 topography and morphology codes used to define the cohort and CCI codes used to define surgery. Online only.

ICD-O-3 topography codes used to define esophageal and gastroesophageal junction cancer:

C15.0-C15.9, C16.0-C16.2, C16.5, C16.8, and C16.9.

ICD-O-3 morphology codes used to define adenocarcinoma and squamous cell carcinoma:

| Adenocarcinoma | Squamous Cell Carcinoma |
| --- | --- |
| 81403, 81413, 81423, 81433, 81443, 81453, 82003, 82103, 82113, 82143, 82503, 82553, 82603, 82613, 82623, 82633, 82903, 83103, 83233, 83803, 84013, 84603, 84803, 84813, 84823, 84903, 85003, 85423, 85703, 85723, 85743, 85763. | 80513, 80523, 80703, 80713, 80723, 80743, 80763, 80833, 85603, 89803. |

Surgical resection was defined using the Canadian Classification of Health Interventions codes for esophagectomy or gastrectomy (CCI codes 1.NA.87-92.^^ and 1.NF.89-90.^^).

**Supplemental Data 2, Table:** Data sources for the study. Online only.

| Database | Information |
| --- | --- |
| Ontario Cancer Registry | Cancer information including histology |
| Ontario Health Insurance Plan Database | Inpatient and outpatient physician billing data; includes information on diagnoses and services provided |
| Canadian Institute for Health Information Discharge Abstract Database (CIHI-DAD) | Information from hospital admissions including diagnoses and receipt of esophagectomy or gastrectomy; mandatory reporting system for all institutions in Ontario |
| Registered Persons Database (RPDB) | Derived from all administrative data sources and provides demographic data including median neighbourhood income quintile, vital status, and details such as date of last contact with the healthcare system |
